# Supplementary material for: Resolving the full spectrum of human genome variation using Linked-Reads
Source: Genome Res. 2019 Apr;29(4):635–45. doi: 10.1101/gr.234443.118 (PMC6442396; doi:10.1101/gr.234443.118)
Supplement: Supplemental Material [file supp_gr.234443.118_Supplemental_longranger-2.2.zip › longranger-2.2.tar.bz2!/lr2.2-public/lib/stan/cmdstan/stan/doc/api/html/pages.html]

Stan: Related Pages


|  |  |
| --- | --- |
|  | Stan  2.5.0  probability, sampling & optimization |


- Main Page
- Related Pages
- Namespaces
- Classes
- Files


All Classes Namespaces Files Functions Variables Typedefs Enumerations Enumerator Friends Macros Pages

Related Pages

Here is a list of all related documentation pages:

|  |  |
| --- | --- |
| Error Handling Policies | Describe the error handling policies for stan::prob here |


---

[ Stan Home Page ]

*© 2011–2014,
Stan Development Team.*
